# Comparative Analysis of Conventional and Focused Data Augmentation Methods in Rib Fracture Detection in CT Images

**DOI:** 10.3390/diagnostics15151938

**Published:** 2025-08-01

**Authors:** Mehmet Çağrı Göktekin, Evrim Gül, Feyza Aksu, Yeliz Gül, Metehan Özen, Yusuf Salik, Merve Kesim Önal, Engin Avci

**Affiliations:** 1 Department of Emergency Medicine, Firat University, 23119 Elazig, Turkey; mc.goktekin@firat.edu.tr (M.Ç.G.); egul@firat.edu.tr (E.G.); 2 Department of Anatomy, Firat University, 23119 Elazig, Turkey; feyzaaksu@firat.edu.tr; 3 Department of Radiology, Elazig Fethi Sekin City Hospital, 23280 Elazig, Turkey; yeliz_gul78@hotmail.com; 4 Institute of Science, Firat University, 23119 Elazig, Turkey; meteozen688@gmail.com (M.Ö.); salikyusuf62@gmail.com (Y.S.); merve.kesim@ozal.edu.tr (M.K.Ö.); 5 Department of Computer Engineering, Faculty of Engineering and Natural Sciences, Malatya Turgut Ozal University, 44900 Malatya, Turkey; 6 Department of Software Engineering, Faculty of Technology, Firat University, 23119 Elazig, Turkey

**Keywords:** rib fracture, YOLOv8, CT images, data augmentation, focused augmentation

## Abstract

**Background/Objectives**: Rib fracture detection holds critical importance in the field of medical image processing. **Methods**: In this study, two different data augmentation methods, traditional data augmentation (Albumentations) and focused data augmentation (focused augmentation), were compared using computed tomography (CT) images for the detection of rib fractures on YOLOv8n, YOLOv8s, and YOLOv8m models. While the traditional data augmentation method applies general transformations to the entire image, the focused data augmentation method performs specific transformations by targeting only the fracture regions. **Results**: The model performance was evaluated using the Precision, Recall, mAP@50, and mAP@50–95 metrics. The findings revealed that the focused data augmentation method achieved superior performance in certain metrics. Specifically, analysis on the YOLOv8s model showed that the focused data augmentation method increased the mAP@50 value by 2.18%, reaching 0.9412, and improved the recall value for fracture detection by 5.70%, reaching 0.8766. On the other hand, the traditional data augmentation method achieved better results in overall precision metrics with the YOLOv8m model and provided a slight advantage in the mAP@50 value. **Conclusions**: The study indicates that focused data augmentation can contribute to achieving more reliable and accurate results in medical imaging applications.

## 1. Introduction

Rib fractures are frequently seen after high-energy traumas such as traffic accidents and falls from heights, and if not detected correctly in time, they can cause vital complications such as pneumothorax and hemothorax. Rib fractures are among the most common injuries in the chest region. In clinical settings, the fractured edge of the rib can irritate the intercostal nerve and cause localized pain [1]. Today, diagnostic methods for rib fractures are mainly based on X-ray and computerized tomography (CT) examinations. However, X-ray examinations may be insufficient in detecting small, hidden, or non-obvious fractures and may cause them to be overlooked [2].

Chest X-ray is a frequently used method to diagnose rib injuries. However, the general structure of the ribs is arranged in a low and high anterior ring shape, and the bone cortex has a flat structure. Therefore, most of the ribs cannot be viewed close to the film during the shooting. This makes it difficult to detect some fractures without obvious displacement and can cause especially thin fractures and fractures in special areas to be overlooked. The detection of fractures is a complex process, and the fact that especially small and difficult-to-see fractures are overlooked reveals the limitations of traditional analysis methods. This situation increases the need for automatic and high-accuracy algorithms in rib fracture detection, which is an important research topic in the field of medical image processing. Among these, YOLO models are widely preferred in medical image processing studies due to their fast and precise object detection capabilities [3].

The successful application of machine learning models usually depends on large, high-quality, and annotated datasets. However, the process of creating such datasets is quite time-consuming and costly. Access to sufficient data sources is even more limited, especially in areas that require expertise such as medical imaging. Therefore, data augmentation methods stand out as an important solution to overcome the limited data problem. Data augmentation aims to both increase the amount of data and improve model performance by diversifying the training data [4]. Traditional data augmentation techniques such as flipping, rotation, and scaling are commonly used to expand training datasets and enhance model generalization. While effective in many applications, these global transformations may not adequately preserve local contextual features, which are critical for detecting subtle anomalies in medical images [5]. Albumentations data augmentation library was designed to overcome these limitations. Albumentations, which has a user-friendly interface and fast processing capacity, supports not only basic transformations but also complex image operations. This flexibility allows the library to be easily adapted to different computer vision tasks and offers a significant advantage in sensitive areas such as medical imaging [6].

All these data augmentation methods are usually applied to the entire image, and typically, only class labels are considered. However, when there are multiple objects in an image, augmentation strategies that provide sensitivity to local context become important. In this study, a new object-oriented data augmentation method is proposed on CT images, which are frequently used for the detection of rib fractures. The method is compared with traditional data augmentation methods in the literature, and its contribution to the automatic detection of rib fractures is evaluated. Yang et al. investigated the accuracy and speed of a deep learning-based system they developed for the detection and classification of rib fractures in CT images. The system provided faster results and higher accuracy for some types of fractures compared to radiologists. In addition, a second model for the detection and classification of fractures showed high accuracy in distinguishing old and new fractures (87.63%). Such studies constitute an important step in the integration of automatic diagnostic tools into clinical processes [3]. Jin et al. demonstrated the potential of convolutional neural networks (CNNs) in this field by developing FracNet, a deep learning-enabled system that demonstrated high accuracy in detecting and segmenting rib fractures from CT scans [7]. Zhou et al. applied a convolutional neural network model that achieved high sensitivity and specificity to cross-modal data (clinical information and CT images) for detecting and classifying fresh, healing, and old fractures based on CT images [8]. Li et al. [9] developed a YOLO-based model that detects rib fractures from CT images and classifies them according to displacement severity. Trained with data collected from two centers, the model showed high success in internal and external tests with an F1 score of 0.948 and an mAP50 value of 0.972. Yao et al. [10] developed a three-stage deep learning model that includes rib segmentation, localization, and classification to detect rib fractures in chest CT images using a three-step algorithm. The model, trained on a dataset of 1707 patients, demonstrated high accuracy, especially when used in conjunction with radiologists.

Data augmentation techniques are generally divided into two categories: geometric transformations and photometric (color space) transformations. Geometric transformations enhance data diversity by altering the spatial properties of images, such as position, scale, and orientation. Taylor and Nitschke [11] investigated the effects of transformations such as flipping, random rotation between −30° and 30°, and cropping on model performance. Their study demonstrated that these techniques improve the training process. Similarly, Kang et. al. [12] proposed a data augmentation method called PatchShuffle Regularization. In this method, pixel values within an nxn time sliding window are randomly shuffled. Experiments conducted on the CIFAR-10 dataset reported a reduction in the error rate from 6.33% to 5.66%. On the other hand, photometric transformations aim to enhance data diversity by modifying the color components of images. These transformations include color space conversions (RGB, HSV, YUV, etc.), brightness and contrast adjustments, and random color manipulation (color jittering). Chatfield et al. [13] demonstrated in their experiments on the ImageNet and PASCAL VOC [14] datasets that grayscale images achieved approximately 3% lower classification accuracy compared to RGB images. Jurio et al. [15] compared the effects of different color spaces, such as RGB, YUV, CMY, and HSV, on image segmentation. While color space transformations offer certain advantages, they also come with some drawbacks. For instance, they can increase memory usage, introduce additional computational costs, and extend training time. Moreover, color modifications may lead to undesirable outcomes in specific applications. In tasks like image sentiment analysis, color alterations can negatively impact the model’s prediction performance. In particular, when the red color is crucial for distinguishing between blood, paint, or other liquids such as water, incorrect color transformations may hinder the model’s ability to make this distinction [16]. Some researchers have developed new approaches to enable the model to automatically learn color transformations. Karargyris [17] introduced a model called the Color Space Transformation Network, proposing a method that allows the neural network to learn the optimal color space transformation. This approach demonstrated faster convergence (8 epochs vs. 15 epochs) and higher accuracy (68% vs. 50%) in experiments conducted on the CIFAR-10 dataset. The system learns the optimal color transformation by creating a 3 × 3 transformation matrix applied to the pixel values of each image. In summary, both geometric and photometric transformations play a significant role within data augmentation strategies. However, the selection of transformations should be made carefully, depending on the dataset to be used and the model’s targeted performance metrics. Geometric transformations help the model become more robust to spatial changes, while color space transformations can provide either advantages or disadvantages for specific applications. Studies have shown that when these two types of transformations are used in a balanced manner, they can enhance model performance.

Bounding box-based data augmentation methods are widely used in the training of object detection and segmentation models and have been developed specifically to address the problem of data imbalance. Compared to traditional data augmentation techniques, these methods provide more targeted data augmentation by focusing only on specific objects and, thus, contribute to increasing the generalization capacity of the model. Various approaches have been proposed in the literature in this context. Ghiasi et al. [18] proposed the copy-paste augmentation method, aiming to increase data diversity by copying objects to different images, and achieved a 0.6% improvement in mask AP and a 1.5% improvement in box AP on the COCO dataset. Dwibedi et al. [19] developed the cut-paste augmentation method, aiming to increase the diversity of the training dataset by randomly cutting and pasting objects, and achieved an accuracy improvement of 2–4% on certain datasets. Hendrycks et al. [20] presented the AugMix method, combining bounding box transformations with mixed data augmentation techniques to strengthen the generalization capacity of the model, and achieved a 4% improvement in the robustness scores of the models on the ImageNet-C dataset. Cubuk et al. [21] developed the auto augment method, which automatically learns different data augmentation policies and applies transformations at the bounding box level, resulting in a 1.5–2% mAP improvement on the COCO dataset. The common goal of these methods is to detect small objects, eliminate data imbalance, and increase model generalization capacity. In addition to general data augmentation strategies, some studies have explored augmentation techniques specifically tailored for fracture detection in medical imaging. Tsai et al. [22] developed a YOLOv5-based model using data augmentation and image enhancement to detect rib fractures on frontal and oblique chest X-rays. This approach improves the model’s ability to recognize fractured ribs in both views, addressing data limitations. Ju and Cai [23] developed a YOLOv8 deep learning model for fracture detection in X-ray images for wrist trauma. Trained using data augmentation methods with images from the GRAZPEDWRI DX dataset, the model outperformed previous YOLOv7-based studies and achieved mAP50 at the SOTA level. Erne et al. [24] developed a DCNN based on Med3D for detecting acetabular fractures from CT scans, using bone area extraction, data augmentation, and global average pooling to improve detection accuracy from 58.8% to 82.8% despite limited training data. Fedora and Gunawan [25] developed Albument-NAS by integrating One Shot Detector with image augmentation techniques, achieving notable improvements in fracture detection accuracy, precision, and recall compared to previous models. El Kojok et al. [26] used synthetic data generated by VAE-GAN models to improve the detection of vertebral compression fractures in chest CT scans. Training with both real and generated images increased classification accuracy by 16%, highlighting the effectiveness of generative augmentation. The focused augmentation method we propose in our study has a similar perspective to these methods but aims to further increase the sensitivity of the model on small and hard-to-detect objects by applying sensitive data augmentation in specific regions.

### Contribution and Novelty

- Unlike conventional data augmentation methods commonly used in literature, this study proposes a region-specific focused data augmentation technique that applies transformations exclusively to fracture areas.
- The method leverages Gaussian filtering and speckle noise addition targeted on annotated fracture regions, aiming to enhance the model’s detection capability for small and complex patterns.
- The study provides one of the first systematic comparisons of three different YOLOv8 models (YOLOv8n, YOLOv8s, and YOLOv8m) under different data augmentation strategies.
- The experiments were conducted on a newly collected CT imaging dataset obtained from a real clinical environment, which had not been used in previous studies.
- The results demonstrated that focused data augmentation could outperform traditional augmentation methods, especially in detecting small fractures.
- The study also highlighted the necessity of future evaluations on larger models (e.g., YOLOv8L, YOLOv8X) and multi-center CT datasets, opening new avenues for further research.

## 2. Materials and Methods

In this section, the dataset, data augmentation methods, and implementation details of the proposed model are presented.

All experiments were conducted on a workstation equipped with an Intel^®^ Core™ i7 13th Gen 3.70 GHz processor, 16 GB RAM, and NVIDIA^®^ GeForce RTX 4060 GPU with 8 GB VRAM (NVIDIA Corporation, Santa Clara, CA, USA). The software environment included Python 3.10 (Python Software Foundation, Wilmington, DE, USA), CUDA Toolkit 12.1, cuDNN version 90100, and PyTorch 2.5.1+cu121 (Meta Platforms Inc., Menlo Park, CA, USA). All experiments were performed on Microsoft Windows 11 Pro.

### 2.1. Dataset

**Figure 1:**
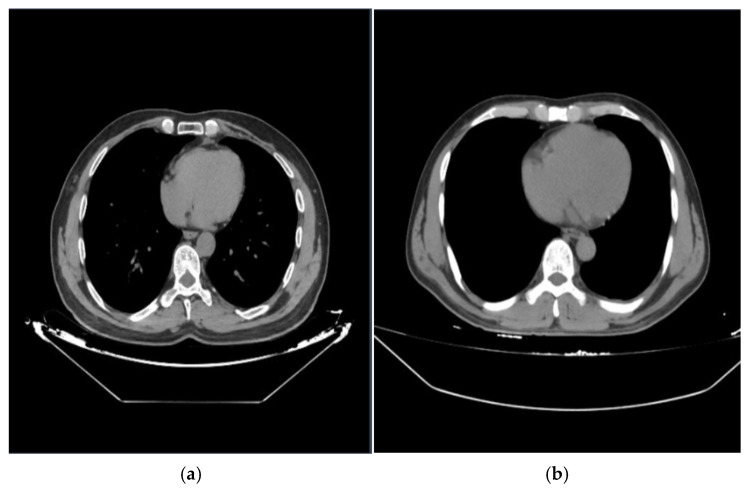
Example of a general CT image with multiple fractures: (**a**) random sample 1 from the dataset; (**b**) random sample 2 from the dataset.

**Figure 2:**
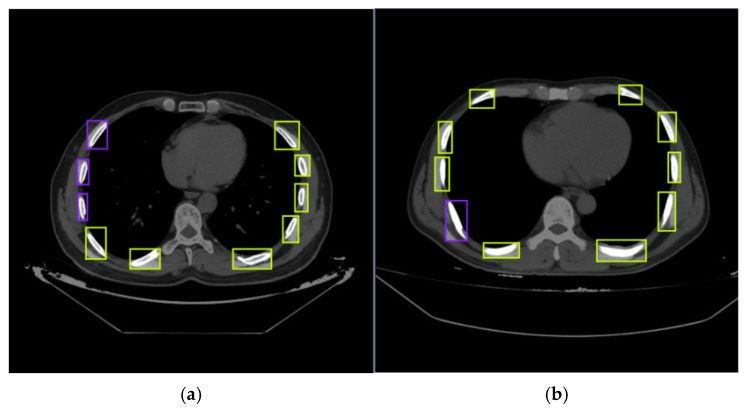
CT images labeled according to fractured and non-fractured areas: Green boxes indicate non-fractured regions, and purple boxes indicate fractured regions: (**a**) random sample 1 from the dataset; (**b**) random sample 2 from the dataset.

The dataset used in this study consists of 2000 computed tomography (CT) images obtained from the Emergency Medicine Department of the Faculty of Medicine at Fırat University (Elazig, Turkey) and anonymized for research purposes. The dataset includes two primary classes: fractured and non-fractured regions, which were carefully annotated by radiologists. Each CT image has a resolution of 640 × 640 pixels. The dataset was then randomly divided into three subsets: 70% for training, 20% for validation, and 10% for testing. Sample images from the dataset include cases with multiple fractured and non-fractured labels, reflecting its overall structure, as shown in Figure 1. The annotated versions of the fractured and non-fractured regions within these images are presented in Figure 2.

**Figure 3:**
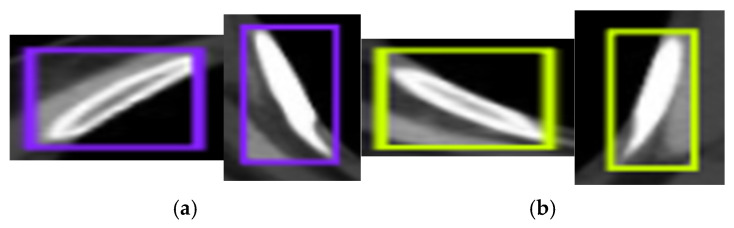
Examples of labeled regions on CT images: (**a**) labeled representation of fractured regions and (**b**) labeled representation of non-fractured regions.

The number of classes within each image in the dataset varies, with non-fractured regions being more prevalent. This indicates that the dataset has a heterogeneous structure. To address this class imbalance, particularly in the fractured class, the use of data augmentation techniques has become necessary. Sample representations of the fractured and non-fractured regions, indicated in the labels in Figure 2, are illustrated in Figure 3.

### 2.2. Data Augmentation Methods

In recent years, deep learning-based data augmentation methods have played an important role in medical imaging. Data augmentation helps to address overfitting problems, which are often linked to the limited size and diversity of the training dataset.. This is done with the assumption that more information can be extracted from the original dataset through augmentations. These augmentations artificially increase the size of the training dataset through data skewing or oversampling [5]. In particular, deep generative models such as generative adversarial networks (GANs) and diffusion models stand out as effective tools that provide higher accuracy and generalization capacity by increasing data diversity [27].

Two different data augmentation strategies were applied to enhance the model’s performance in detecting rib fractures: traditional data augmentation and focused data augmentation. Albumentations were preferred for the traditional data augmentation method. Albumentations offers a rich variety of data augmentation techniques for tasks such as image classification, segmentation, and object detection, and performs optimized transformations that perform better than alternative methods. General transformations were applied to all images in the training dataset, including random rotation (±30°), brightness and contrast enhancement, and blurring. With this data augmentation strategy, the size of the dataset was doubled, increasing the generalization ability of the model and preventing over-learning.

**Figure 4:**
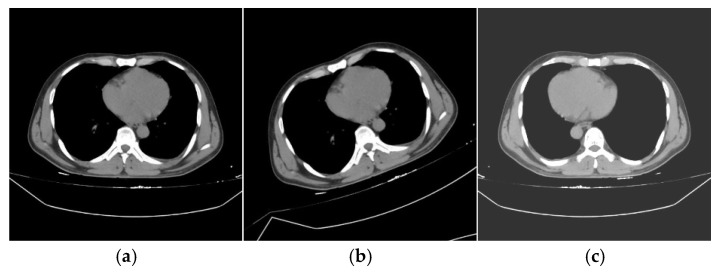
Examples of traditional data augmentation applied using Albumentations: (**a**) original rib fracture image, (**b**) image with random rotation (±30°), and (**c**) image with brightness and contrast applied.

Examples of the traditional data augmentation techniques applied to the training dataset using Albumentations are presented in Figure 4. The figure illustrates how general transformations, such as random rotation, brightness and contrast adjustments, and blurring, alter the original images. Subfigure (a) shows the original rib fracture image before any augmentation, while subfigures (b) through (c) demonstrate the effects of different transformations.

In this study, the proposed method aims to augment data by focusing on the fracture regions in the images in the training dataset, unlike traditional data augmentation methods. To achieve this, bounding box information is used to define the fracture regions, and transformations are applied only to these areas. This approach is designed to increase the ability of the model to recognize fracture regions and improve detection performance. The focused data augmentation technique is developed based on the object-focused image data augmentation (OFIDA) model [28] in the literature, and various and carefully adapted augmentations are applied to the fracture regions. By adopting this method, the model is protected from irrelevant areas and is made to focus only on the target regions, thus contributing to a more efficient and effective training process.

To enable the model to better recognize these specific regions, the transformations applied to the fractured regions defined by the bounding box were selected as speckle noise and Gaussian filter. The application of speckle noise simulates varying imaging conditions, such as low-quality or distorted images, and helps the model generalize better by learning to detect fractures under different scenarios. Following the addition of noise, the fractured regions were smoothed using a Gaussian blur filter, enabling the model to capture complex patterns and structures within the fractured regions more efficiently. The overall transformation process can be expressed as:

(1) $$I^{\prime }=clip\left(I+I\cdot N\left(0,\sigma^{2}\right),0,\;255\right)$$

where $I^{\prime }$ denotes the transformed fracture region, I is original pixel value, $N(0,\;\sigma^{2})$ represents the Gaussian noise with zero mean and variance $\sigma^{2}$, and clip(.) confines the resulting pixel values within the range [0, 255] to maintain valid image intensities.

The noise-added fractured regions are smoothed with the Gaussian blur filter, increasing the capacity of the model to learn complex patterns in fractured regions. Gaussian blurring is defined by the following equation:

(2) $$I^{\prime \prime }\left(x,y\right)=\sum_{i=-k}^{k}\sum_{j=-k}^{k}I^{\prime }\left(x+i,y+j\right)\cdot G\left(i,j\right)$$

where G(i,j) is the Gaussian weight, k is half the filter size (e.g., *k* = 2 for 5 × 5), x and y are the center coordinates of the pixel to be blurred, and i and j are the surrounding pixel offsets in the filter size.

**Figure 5:**
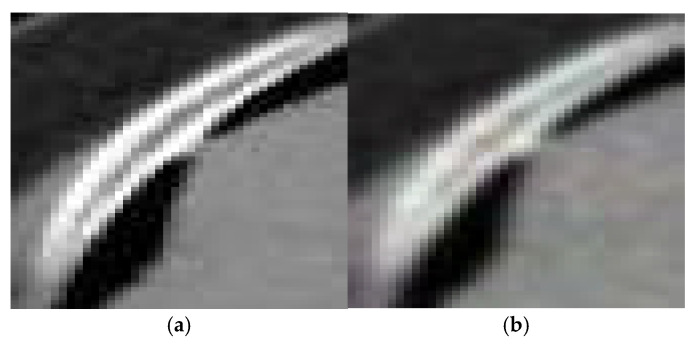
(**a**) Original fracture region and (**b**) fracture region with added noise and Gaussian blurring applied.

The original fracture region is shown in Figure 5a. In Figure 5b, the focused augmentation method is applied to add noise to the region, and the Gaussian blur is applied. These transformations aim to increase the contrast of the fracture region with the surrounding tissues.

**Figure 6:**
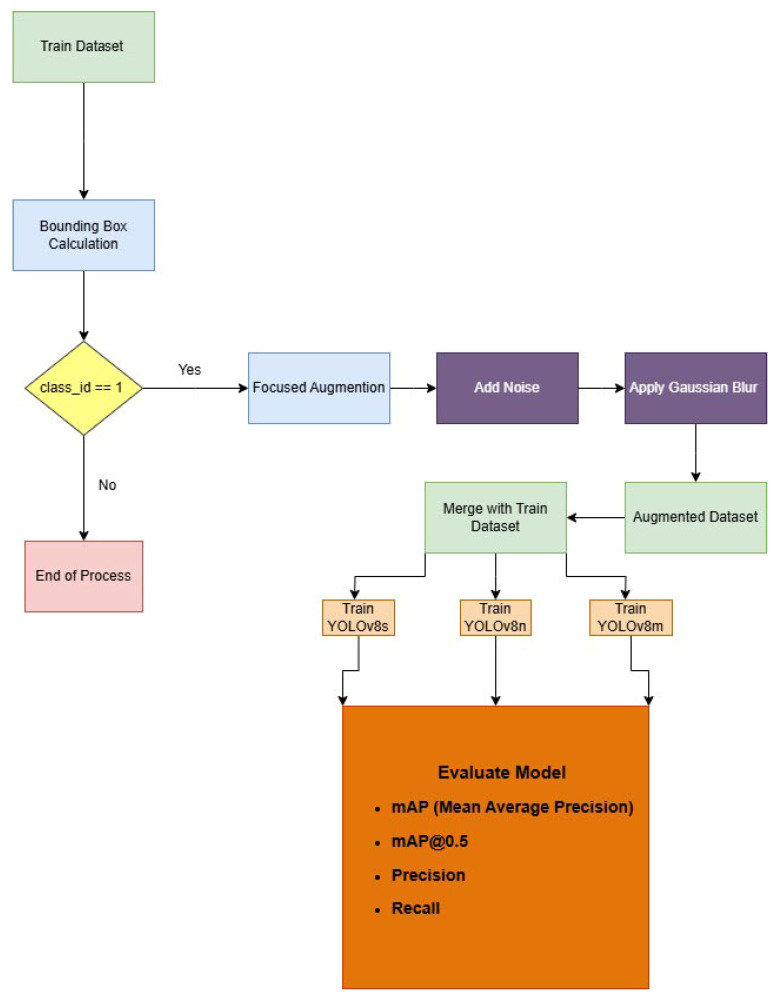
Process flow of focused data augmentation.

The process flow summarizing the focused data augmentation technique is shown in Figure 6. The flow begins with the identification of fracture regions using bounding box annotations, continued by the application of speckle noise and Gaussian blurring. The augmented dataset is then merged with the original training set, and models with varying capacities (YOLOv8n, YOLOv8s, and YOLOv8m) are trained to evaluate the effects of the proposed augmentation technique.

Focused data augmentation offers a more specific approach compared to traditional methods that apply general transformations to the entire image. This method provides local improvements for specific needs by only applying to fracture regions. This approach has significant potential, especially for regions with small fractures or requiring careful analysis. By focusing on critical areas, it significantly increases the sensitivity and accuracy of the model. Such improvements provide great benefits by effectively capturing subtle and difficult patterns in areas requiring high accuracy.

The key distinction of the proposed focused data augmentation method lies in its region-specific application of transformations exclusively within annotated fracture areas. Unlike conventional methods that either globally apply transformations or apply them to pre-defined ROI masks, our approach systematically targets only the fracture regions identified by bounding boxes. This ensures that the model intensively learns the characteristic features of fractures without being affected by irrelevant areas, enhancing its detection capability on small and complex patterns.

### 2.3. Classification Method

To evaluate the impact of different data augmentation strategies on model performance, models with varying architectural complexities and capacities are essential. This allows for a comprehensive analysis of how augmentation affects detection accuracy and generalization across models with different learning capacities [29]. In this study, three different models belonging to the YOLOv8 family, which is supervised learning (YOLOv8n, YOLOv8s, and YOLOv8m), were used. These models have increasing levels of complexity and capacity and have been used to test the effects of different data augmentation strategies. Table 1 compares the variants used from the YOLO v8 family [30].

The YOLOv8n (Nano), YOLOv8s (Small), and YOLOv8m (Medium) models address different needs, starting from low computational costs to models with higher computational capacity. The performance of the model was optimized by using certain hyperparameters during the model training process. In order to standardize the dimensions of the CT images and achieve high accuracy, the input size was determined as 640 × 640 pixels. To ensure that the model had sufficient training time and to increase its generalization ability, 60 epochs were used. A batch size of 16 was preferred considering the GPU memory limits and computational costs. A learning rate of 0.001 was determined to ensure the stability of the model and to achieve a suitable learning rate. The AdamW optimization algorithm was preferred to ensure that the training process progressed faster and more steadily.

The performance of the models was analyzed using various evaluation metrics. Precision expresses the ratio of correctly detected fractures to total predictions and shows the ability of the model to reduce the false positive rate. Recall measures the sensitivity of the model by determining how many of the true fractures were correctly detected. mAP@50 is a metric calculated using a threshold value of 50% intersection over union (IoU) to evaluate detection accuracy. mAP@50–95 provides a more comprehensive analysis of the overall detection ability by evaluating the average performance of the model at IoU threshold values ranging from 50% to 95%.

The testing process was conducted to evaluate the final performance of the models after training. In this process, the test set was used to measure the generalization capacity and accuracy of the models. The test results allowed for a detailed analysis of the effects of the data augmentation strategies.

## 3. Results

In this study, the performance of three models belonging to the YOLOv8 family (YOLOv8n, YOLOv8s, and YOLOv8m) on rib fracture detection was compared using traditional (Albumentations) and focused (focused augmentation) data augmentation methods. Performance evaluation was conducted on metrics such as mAP@50, mAP@50–95, Precision, and Recall. The findings of the study clearly reveal how data augmentation strategies affect the accuracy and generalization capacity of deep learning models. The performance results of the models trained with the focused augmentation and Albumentations methods is presented in detail in Table 2 and Table 3.

The focused data augmentation method provided significant improvements in precision and recall values in the fracture class, making significant contributions to the model performance. The most remarkable results of this method were observed on the YOLOv8s model. The YOLOv8s model reached 0.941 with a 2.18% increase in mAP@50 using focused data augmentation. In addition, the recall value increased by 5.70% to 0.877. These improvements reveal the effectiveness of the method, especially in detecting small and complex fractures. Focused augmentation has increased the sensitivity of the model in these critical areas thanks to the transformations achieved by focusing only on the fracture areas. The fact that this target-oriented approach can successfully identify small and difficult-to-detect fractures is considered an important innovation in the field of medical image processing. Albumentations, a traditional data augmentation method, has stood out as a method with a wider generalization capacity. It has shown the highest success, especially on the YOLOv8m model. The YOLOv8m model reached 0.924 with a 1.10% increase in mAP@50 value with Albumentations and achieved superiority in precision values. The general transformations applied to the entire image in this method increased the generalization capacity of the model and provided a more balanced performance on large datasets. Albumentations, thanks to its variety of different transformations and flexibility, allows successful results to be obtained, especially in large datasets with high generalization requirements. Its wide-ranging generalization capacity increases the potential of the method in medical imaging and other computer vision areas. The YOLOv8n model stands out with its lightweight structure and low computational cost, providing fast training time. Using the focused augmentation method, the mAP@50 value increased by 0.99% to 0.921. Improvements of 4.37% and 0.51% were observed in the precision and recall metrics, respectively. The YOLOv8s model was evaluated as the most balanced model in terms of performance. In this model, the precision value decreased by 5.73% in the fracture class with the focused augmentation method, while the recall value increased by 5.70%. This situation reveals the potential of the model to be optimized for certain classes. The YOLOv8m model, on the other hand, outperformed traditional data augmentation methods and reached 0.924 with a 1.10% increase in the mAP@50 value with the Albumentations method.

However, when the focused augmentation method was used, decreases in precision and recall values were observed, indicating that the model required special optimization for the fractured class.

**Figure 7:**
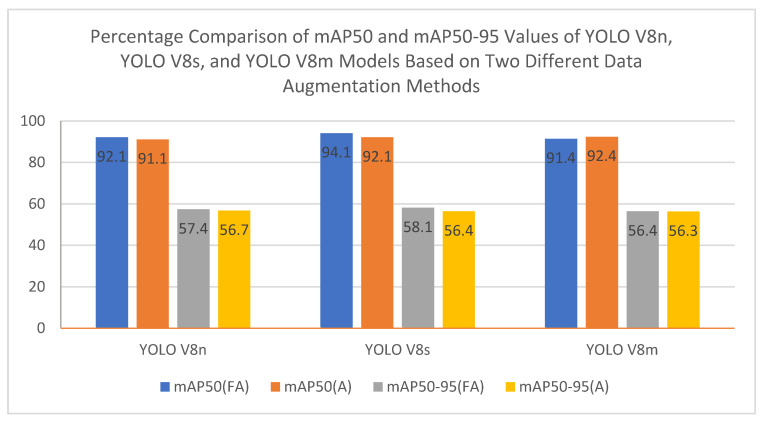
Percentage comparison of focused augmentation and Albumentations across mAP metrics.

**Figure 8:**
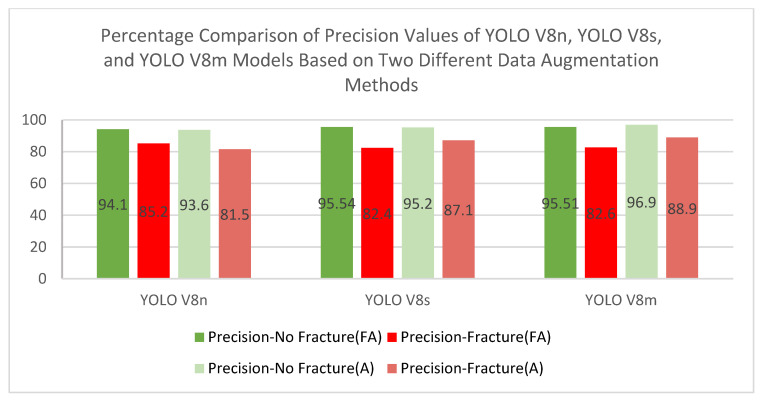
Percentage comparison of precision values of all models.

**Figure 9:**
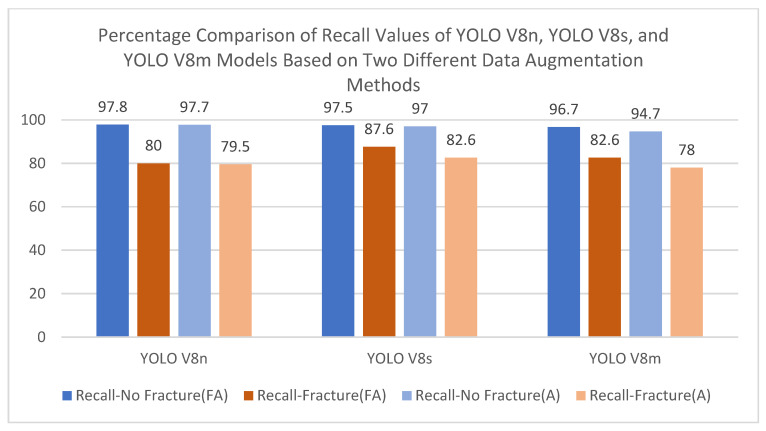
Percentage comparison of recall values of all models.

Comprehensive visualizations of model performance metrics across different augmentation strategies are provided in Figure 7, Figure 8 and Figure 9. Figure 7 illustrates the percentage comparison of mAP@50 and mAP@50–95 values for YOLOv8 models when focused augmentation (FA) and Albumentations (A) are applied, highlighting the differences in detection accuracy. Figure 8 presents the precision values for fractured and non-fractured regions, showing how accurately the models distinguish between classes under varying augmentation methods. Lastly, Figure 9 focuses on the recall values, demonstrating the model’s sensitivity to identifying fractured and non-fractured regions across all models and augmentation techniques.

**Figure 10:**
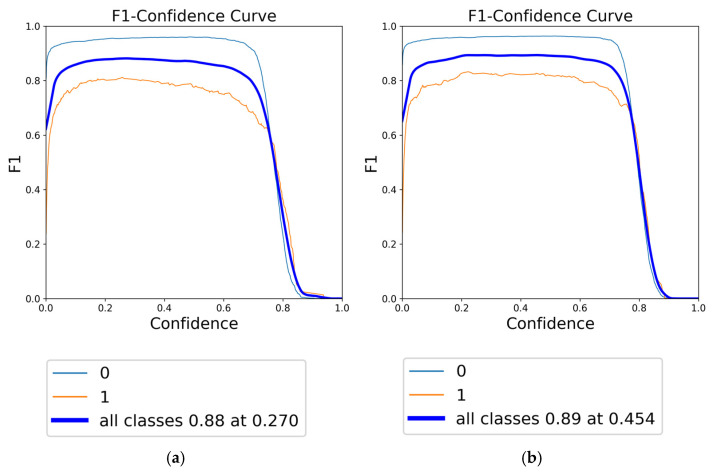
Comparison of F1-confidence curves for YOLOv8n using different data augmentation strategies: (**a**) Albumentations and (**b**) focused augmentation.

**Figure 11:**
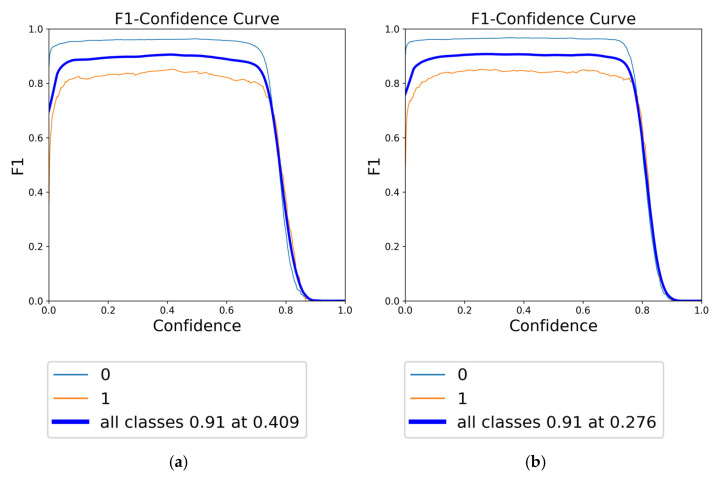
Comparison of F1-confidence curves for YOLOv8s using different data augmentation strategies: (**a**) Albumentations and (**b**) focused augmentation.

**Figure 12:**
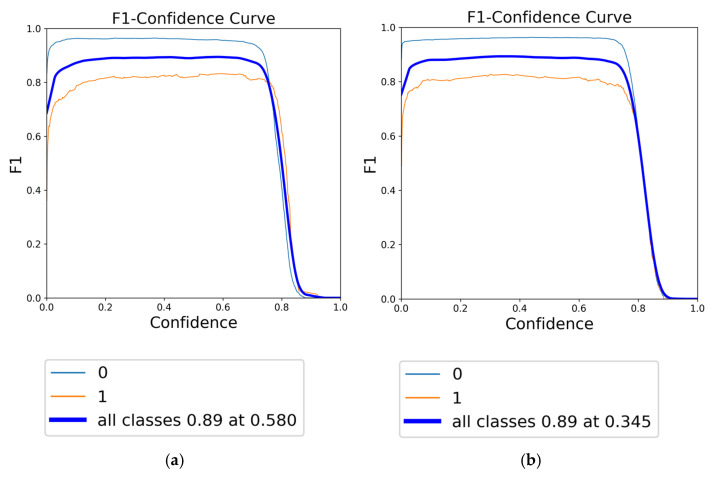
Comparison of F1-confidence curves for YOLOv8m using different data augmentation strategies: (**a**) Albumentations and (**b**) focused augmentation.

The F1 score, a harmonic mean of precision and recall, is a crucial metric for evaluating the overall accuracy and robustness of a model. In this context, the F1-confidence curves for YOLOv8 models under different augmentation strategies are presented in Figure 10, Figure 11 and Figure 12. Figure 10 illustrates the F1-confidence curve for the YOLOv8n model, demonstrating the comparative effects of focused augmentation and Albumentations on the detection of fracture and non-fracture regions. Figure 11 presents the F1-confidence curve for the YOLOv8s model, highlighting its stable and balanced performance across various confidence thresholds and augmentation strategies. Figure 12 displays the F1-confidence curve for the YOLOv8m model, emphasizing how different augmentation methods influence the model’s detection capability and generalization in diverse scenarios.

**Figure 13:**
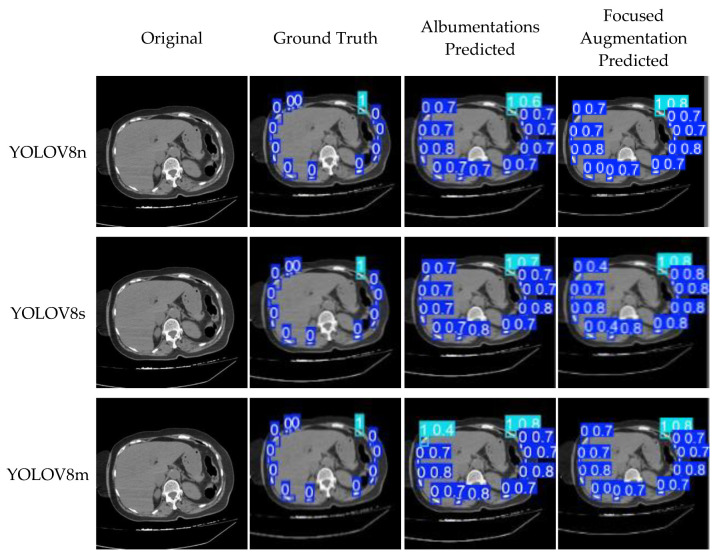
Visual comparison of detection results of YOLOv8 models (n, s, m) using Albumentations and focused augmentation.

Figure 13 illustrates visual comparisons for the three YOLOv8 models using both the Albumentations and focused augmentation strategies applied to a sample image from the test dataset. The prediction outputs are formatted as (class, confidence), where the class label indicates the fracture presence (1: fracture, 0: no fracture), and the confidence score shows the model’s prediction certainty. As shown in the figure, the focused augmentation method allows for more accurate and confident fracture detection, particularly for cases with subtle or overlapping rib structures.

## 4. Discussion

This study comprehensively examined the performance of the YOLOv8n, YOLOv8s, and YOLOv8m models in rib fracture detection in the context of traditional (Albumentations) and focused (focused augmentation) data augmentation methods. The results show that data augmentation methods significantly affect the accuracy and generalization capacity of deep learning models.

The focused data augmentation method provided substantial improvements, particularly for smaller models and challenging fracture types. For example, the YOLOv8n model stands out with its lightweight structure and low computational cost. This model provided 0.99% improvement in mAP@50 value and 0.52% and 0.51% improvement in precision and recall metrics, respectively, with the focused data augmentation method. The YOLOv8s model increased by 2.18% in mAP@50 value and 5.70% in recall value in the fractured class with the focused augmentation method. The YOLOv8m model, which has a more complex structure, showed higher mAP@50 with traditional augmentation (+1.10%). This specific outcome stems from the interaction between model capacity, dataset characteristics, and augmentation strategy: Our analysis reveals that the higher parametric capacity of V8m, combined with the limited dataset size (2000 CT scans with class imbalance), may have constrained its ability to fully generalize from focused augmentations. This aligns with scaling law principles, where larger models exhibit greater sensitivity to data limitations during augmentation. Importantly, the same model showed improvement in a more stringent mAP50–95 metric (+0.21%) with focused augmentation, indicating enhanced precision in fracture localization. These results have shown that data augmentation has significant benefits in fracture detection, similar to the studies by El Kojok Z et al. [26] and Ju and Cai [23], in which they increased the mAP value of the model by augmenting data with various methods.

It has been proven that the focused data augmentation method increases the sensitivity of the model in specific regions by only applying processing to certain areas. This approach is particularly valuable for detecting small or subtle fractures where traditional global augmentation might dilute critical features. The V8m’s response highlights the importance of model-specific augmentation tuning—an optimization opportunity for complex architectures that we will explore in future work.

The focused data augmentation method offers significant advantages in the detection of distinct anatomical structures such as fractures. This method can reduce the margin of error in clinical processes by increasing the detection rate of fractures, especially in low-quality or distorted CT images. In addition, when applied correctly, it can enable the development of decision support systems that will reduce the workload of radiologists and accelerate detection processes.

Although YOLO-based models offer promising performance in real-time detection tasks, clinical applications prioritize diagnostic accuracy and reliability over inference speed. While our experiments were conducted on a high-quality, single-center dataset, the generalizability of the proposed focused augmentation method to diverse clinical scenarios—such as low-dose CT, motion-blurred scans, and multi-institutional datasets with heterogeneous imaging conditions—remains an important consideration. Evaluating the method under such variations would better reflect real-world diagnostic variability and help assess its robustness. This aspect was beyond the scope of the current study but remains a critical direction for future research.

## 5. Conclusions

This study was conducted on a limited dataset obtained from a single institution, which exhibited a homogeneous structure dominated by the non-fracture class. For future work, larger and more diverse datasets with balanced classes are recommended. Furthermore, the integration of advanced data augmentation techniques, such as GAN and diffusion models, could be explored.

It is envisaged that the focused data augmentation method can be effectively applied not only in bone fracture detection but also in the detection of cancerous cells or other pathological conditions.

The focused data augmentation method has provided significant superiority, especially in the identification of small and difficult-to-detect fractures. This study is the first study to systematically compare YOLOv8 models (YOLOv8n, YOLOv8s, and YOLOv8m) for rib fracture detection. One of the innovative aspects of the study is the use of a new dataset obtained from Fırat University Faculty of Medicine, which has not been used before in the literature. The findings show that the focused data augmentation method has potential to be applied in other medical imaging areas.

## Figures and Tables

**Table 1:** Comparison of YOLO v8 models used.

Name	Layers	Parameters	Gradients	GFLOPs
YOLOv8n	195	3,258,454	0	12.0
YOLOv8s	195	11,780,374	0	42.4
YOLOv8m	245	27,223,542	0	110.0

**Table 2:** Performance comparison of focused augmentation and Albumentations across mAP metrics.

Model	Metrics	Focused Augmentation	Albumentations	Comparison
YOLO V8n	mAP50	0.921	0.912	+0.99%
	mAP50–95	0.574	0.568	+1.14%
YOLO V8s	mAP50	0.941	0.921	+2.18%
	mAP50–95	0.582	0.564	+3.06%
YOLO V8m	mAP50	0.914	0.924	−1.10%
	mAP50–95	0.564	0.563	+0.21%

**Table 3:** Performance comparison of focused augmentation and Albumentations across precision and recall metrics.

Model	Metrics	Focused Augmentation		Albumentations		Comparison	
		No Fracture	Fracture	No Fracture	Fracture	No Fracture	Fracture
YOLO V8n	Precision	0.942	0.853	0.937	0.816	+0.52%	+4.37%
	Recall	0.979	0.800	0.978	0.796	+0.13%	+0.51%
YOLO V8s	Precision	0.955	0.824	0.952	0.872	+0.36%	−5.73%
	Recall	0.976	0.877	0.971	0.827	+0.51%	+5.70%
YOLO V8m	Precision	0.955	0.826	0.970	0.889	−1.53%	−7.63%
	Recall	0.968	0.827	0.948	0.780	+2.07%	+5.63%

## Data Availability

The data that support the findings of this study are available on request from the corresponding author.
